# Factors associated with prolonged postoperative length of hospital stay after laparoscopic colorectal cancer resection: a secondary analysis of a randomized controlled trial

**DOI:** 10.1186/s12893-022-01886-4

**Published:** 2022-12-24

**Authors:** Hong Li, Tong-Feng Luo, Nan-Rong Zhang, Li-Zhen Zhang, Xia Huang, San-Qing Jin

**Affiliations:** 1 Department of Anesthesia, The Sixth Affiliated Hospital, Sun Yat-sen University, Guangzhou, China; 2 Guangdong Provincial Key Laboratory of Colorectal and Pelvic Floor Diseases, The Sixth Affiliated Hospital, Sun Yat-sen University, Guangzhou, China

**Keywords:** Postoperative length of hospital stay, Prolongation, Laparoscopy, Colorectal surgery

## Abstract

**Background:**

The postoperative length of hospital stay (PLOS) is an important indicator of surgical quality. We identified perioperative factors that affect prolonged PLOS (PPLOS) after laparoscopic colorectal cancer resection, which is the preferred surgical approach for colorectal cancer, the third most common cancer.

**Methods:**

This study was a secondary analysis of a randomized trial (clinicaltrials.gov ID: NCT03160144) that included 280 patients who underwent laparoscopic colorectal cancer resection. The primary outcome was a PPLOS, defined as a PLOS that was longer than the median PLOS. Baseline, anesthetic, surgical, and postoperative management factors were included in the univariate and multivariate analyses to identify factors influencing PPLOS.

**Results:**

The median PLOS was 10 days, and 117 patients had a PPLOS. We identified six influencing factors for PPLOS: preoperative pulse oxygen saturation < 96% (odds ratio [OR], 3.09 [95% confidence interval (CI) 1.38–6.92]; *P* = 0.006), distant tumor metastasis (OR, 0.34 [95% CI 0.13–0.91]; *P* = 0.031), the Miles procedure or left hemicolectomy (OR, 4.51 [95% CI 1.67–12.18]; *P* = 0.003), perioperative surgical events (OR, 2.44 [95% CI 1.25–4.76]; *P* = 0.009), postoperative albumin infusion (OR, 2.19 [95% CI 1.14–4.19]; *P* = 0.018), and postoperative early ambulation (OR, 0.35 [95% CI 0.18–0.68]; *P* = 0.002). Further stratified analysis showed that postoperative albumin infusion might be a risk factor for PPLOS, even in patients with a preoperative albumin level < 40 g/L (OR, 2.29 [95% CI 0.98–5.34]; *P* = 0.056) or duration of surgery ≥ 3 h (OR, 2.52 [95% CI 1.08–5.87]; *P* = 0.032).

**Conclusions:**

A low preoperative pulse oximetry reading, complex surgical procedures, perioperative surgical events, and postoperative albumin infusion may be risk factors for PPLOS after laparoscopic colorectal cancer resection, whereas distant tumor metastasis and postoperative early ambulation might be protective factors. The association between postoperative albumin infusion, a modifiable factor, and PLOS or clinical outcomes warrants further investigation.

**Supplementary Information:**

The online version contains supplementary material available at 10.1186/s12893-022-01886-4.

## Background

Length of hospital stay (LOS) is at the core of many quality improvement initiatives, as it can reflect the quality of care delivered to patients and is associated with health care costs. Accordingly, a prolonged LOS usually denotes poor quality of medical care and higher medical expenses. In surgical patients, the postoperative LOS (PLOS) is a particularly important component of overall LOS, and shortening the PLOS is one of the goals of enhanced recovery after surgery (ERAS) [1]. Therefore, understanding the factors that influence prolonged PLOS (PPLOS) in patients undergoing surgery is essential.

Colorectal cancer is the third most common cancer [2], and laparoscopic colorectal cancer resection is an important treatment option [3, 4]. The use of ERAS for laparoscopic colorectal cancer resection has become a popular topic recently [5–7]. Previous studies [4, 8–12] have shown that numerous factors were associated with PLOS after abdominal surgery, including age, smoking status, American Society of Anesthesiologists (ASA) physical status, preexisting medical conditions, surgical approach, duration of surgery, intraoperative lung-protective ventilation, surgical team's expertise, blood loss, blood transfusion, surgical complications, early postoperative mobilization, and postoperative nutritional status. However, factors that influence prolonged hospitalization after laparoscopic colorectal cancer resection remain unclear.

The PROtective Ventilation using Open Lung approach Or Not (PROVOLON) trial was a prospective, randomized controlled trial that investigated a lung-protective ventilation strategy on postoperative complications after laparoscopic colorectal cancer resection [13]. The main results of this trial have been previously published [13]. As a secondary outcome of the trial, the median PLOS did not differ between the lung-protective ventilation group and the non-lung-protective ventilation group [13]. In this secondary analysis of the PROVOLON trial findings, we aimed to investigate the influencing factors, particularly modifiable factors, for PPLOS after laparoscopic colorectal cancer resection.

## Methods

### Ethics approval and consent to participate

The PROVOLON trial was a prospective, randomized, controlled trial. The Institutional Ethical Committee of the Sixth Affiliated Hospital, Sun Yat-sen University, Guangzhou, China approved the study on January 9, 2017 (approval number: 2017ZSLYEC-002). The trial was registered at clinicaltrials.gov (ID: NCT03160144). Written informed consent was obtained from all the participants before enrolment. All experiments were performed in accordance with relevant guidelines and regulations. The trial included 280 patients, and the main results have been published [13].

### Study design, setting, and participants

This was a secondary analysis of the PROVOLON trial, which was conducted from January 2017 to October 2018 at the Sixth Affiliated Hospital, Sun Yat-sen University, a comprehensive tertiary hospital in Guangzhou, China known for gastrointestinal surgery.

The inclusion and exclusion criteria for the trial have been previously described [13]. Briefly, patients were eligible for inclusion if they were aged 40 years or older with a body mass index (BMI) < 30 kg/m^2^, had an intermediate-to-high risk of developing postoperative pulmonary complications, and were scheduled for laparoscopic colorectal cancer resection with an expected duration of pneumoperitoneum > 1.5 h. Patients were excluded if they were classified as ASA physical status ≥ IV, had a pulmonary infection or respiratory failure within 1 month, had cardiac failure, had severe chronic obstructive pulmonary disease or pulmonary bullae, had a progressive neuromuscular illness, or were participating in another interventional study. In the original study, patients were randomized to two ventilatory interventions during surgery: low tidal volume ventilation with or without an open-lung strategy (lung-protective ventilation or non-lung-protective ventilation).

### Perioperative management, data extraction and relevant definitions

We obtained detailed preoperative, intraoperative and postoperative data from the PROVOLON trial team.

Postoperative hospital discharge was determined by the attending surgeon based on the discharge criteria after colorectal cancer surgery in our center: (1) the patient was generally in good condition and has basically returned to a normal diet and intestinal function; (2) The body temperature was normal, without positive signs upon abdominal examination, and the results of related laboratory tests were normal or close to normal; and (3) The incision has healed without surgical site infection.

Postoperative management was performed at the attending surgeons’ discretion. Drainage, blood product infusion, hypotension within 3 days after surgery, and other relevant events (including gallstones, kidney stones, intraperitoneal hyperthermic perfusion chemotherapy, and radiofrequency ablation of hepatic metastasis) were obtained from the follow-up data and hospital information system.

Intraoperative surgical events were defined as positive anastomotic leakage test, reoperation or intestinal anastomosis performed twice, duration of non-pneumoperitoneum ≥ 2 h, or abdominal drainage ≥ 200 mL in the post-anesthesia care unit. Early ambulation was defined as the patient being up and ambulatory within 2 days after surgery. The baseline mean arterial pressure (MAP) was defined as the mean value of three preoperative MAP values. Postoperative hypotension was defined as a decrease in MAP > 30% compared with the baseline MAP.

### Outcomes

In the original study, PLOS was a secondary outcome, with no significant difference in the per-protocol population between the two groups [13]. In the present study, we reanalyzed the PLOS in all enrolled patients. The PLOS was defined as the interval between the operation date and the discharge date.

The primary outcome was a PPLOS, defined as a PLOS longer than the median PLOS. According to the definition of PPLOS, the patients were divided into two groups: the control group (patients without a PPLOS) and the case group (patients with a PPLOS).

### Predictors of PPLOS

We assessed several perioperative variables as possible risk factors, including preoperative characteristics (age, sex, BMI, hemoglobin level, white blood cell count, C-reactive protein [CRP] level, albumin level, pulse oxygen saturation [SpO_2_], ASA physical status, cardiocerebrovascular events, chemotherapy, radiotherapy, weight loss ≥ 10%, diabetes mellitus, abdominal surgery history, medical insurance, and tumor TNM stage), intraoperative characteristics (duration of surgery, chief surgeon, surgical approach, additional operation, active enterostomy, intraoperative surgical events, blood loss, lung-protective ventilation, blood product infusion, urine output, lactic acid, hemoglobin level, and glucose level), and postoperative characteristics (hypotension, albumin infusion, early ambulation, physiotherapy, blood product infusion, abdominal drainage, hemoglobin level, albumin level, white blood cell count, high-sensitivity CRP level, and other relevant events).

### Statistical analysis

No power analysis was performed as the incidence of PPLOS in the study population has never been reported in the literature.

Continuous variables are described as mean and standard deviation or as median (25th, 75th percentiles) and were compared using an independent *t* test or the Mann–Whitney *U* test, as appropriate. Categorical variables are reported as numbers (proportions) and were compared using the Fisher exact test.

Variables considered clinically relevant or variables with a *P*-value < 0.1 in univariate analysis were introduced into the multivariate logistic regression model. Numerical or multi-categorical variables were transformed into two- or three-category variables before being entered into the multivariable logistic regression model. For example, we combined similar surgical procedures (e.g., anterior rectal cancer resection [Dixon] and sigmoidectomy) or the procedures with similar trends for PPLOS (e.g., coloanal anastomosis [Parks] and right hemicolectomy) into the same category to form a three-category variable. Variables with missing data were not included in multivariate analysis. Once a final list of variables was constructed, a stepwise logistic regression analysis was performed to examine the relationship between potential influencing factors and PPLOS. The final multivariate model was tested for goodness of fit using the Hosmer–Lemeshow test. The identified influencing factors are presented as odds ratios (ORs) with 95% confidence intervals (CIs).

After identifying the influencing factors for PPLOS, we conducted a further analysis of the association between clinical characteristics and postoperative albumin infusion. Additionally, we conducted a stratified univariate logistic regression analysis (stratification factors: preoperative albumin level and duration of surgery) of the association between postoperative albumin infusion and PPLOS.

Statistical analyses were conducted using SPSS Statistics for Windows, version 17.0 (SPSS Inc., Chicago, IL, USA). All *P*-values were two-sided, and a *P*-value < 0.05 was considered statistically significant.

## Results

**Fig. 1:**
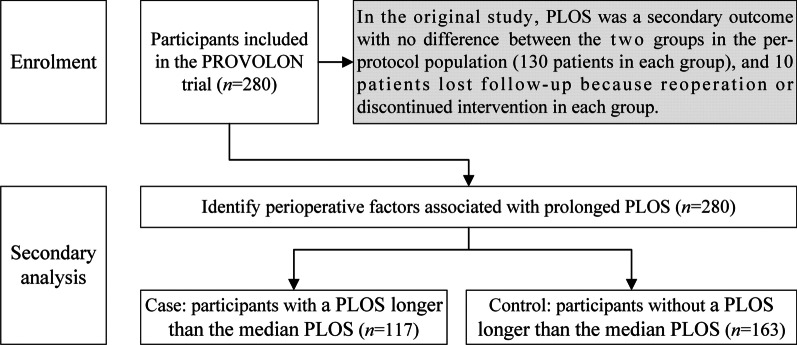
Flow diagram of the study. PROVOLON, PROtective Ventilation using Open Lung strategy Or Not trial; PLOS, postoperative length of stay

According to the median PLOS (10 days), all 280 patients were divided into two groups (Fig. 1): the control group (PLOS ≤ 10 days, 163 cases) and case group (PLOS ≥ 11 days, 117 cases).

As shown in Tables 1, 2, 3, 4, nine possible influencing factors with a *P*-value < 0.1 were introduced into the multivariate logistic regression model, including the preoperative SpO_2_ (*P* = 0.008), distant tumor metastasis (*P* = 0.003), surgical approach (*P* = 0.001), chief surgeon (*P* = 0.052), duration of surgery ≥ 3 h (*P* = 0.05), blood loss ≥ 100 mL (*P* = 0.019), perioperative surgical events (*P* = 0.001), postoperative albumin infusion (*P* = 0.002), and postoperative early ambulation (*P* = 0.001). ASA physical status (*P* = 0.12) and lung-protective ventilation (*P* = 0.15) were also introduced into the multivariate logistic regression model based on clinical experience and the literature[12, 14, 15].

**Table 1:** The associations between baseline characteristics and PPLOS

Characteristic	Total	PLOS ≥ 11 days		*P*-value
		Yes, *n* = 117	No, *n* = 163	
Male sex	200 (71.4)	85 (72.6)	115 (70.6)	0.40
Age (years)	70.3 ± 5.8	70.8 ± 5.4	69.9 ± 6.1	0.23
Age ≥ 75 years	76 (27.1)	32 (27.4)	44 (27.0)	1.00
BMI (kg/m^2^)	22.65 ± 2.77	22.76 ± 2.63	22.57 ± 2.87	0.57
Hemoglobin level (g/L)	121 ± 20	122 ± 22	121 ± 19	0.74
White blood cell count (× 10^9^/L)	6.33 ± 1.99	6.26 ± 1.83	6.39 ± 2.10	0.59
C-reactive protein level (mg/L)	2.71 (1.19, 6.00)	3.26 (1.42, 6.69)	2.38 (1.01, 5.82)	0.10
Albumin level (g/L)	39.3 ± 3.8	39.3 ± 3.6	39.3 ± 3.9	0.98
SpO_2_ (%)	97 (96, 97)	96 (96, 97)	97 (96, 97)	0.008
SpO_2_ < 96%	36 (12.9)	22 (18.8)	14 (8.6)	0.018
ASA physical status (II/III)	226/54	89/28	137/26	0.12
Cardiocerebrovascular events	35 (12.5)	16 (13.7)	19 (11.7)	0.72
Radiotherapy	22 (7.8)	6 (5.1)	16 (9.8)	0.18
Chemotherapy	41 (14.6)	17 (14.5)	24 (14.7)	1.00
Abdominal surgery history	28 (10.0)	13 (11.1)	15 (9.2)	0.687
Weight loss ≥ 10%	61 (21.8)	20 (17.1)	41 (25.2)	0.14
Diabetes mellitus	34 (12.1)	12 (10.3)	22 (13.5)	0.46
T category				0.89
T_3_–T_4_	214 (76.4)	92 (78.6)	122 (74.8)	0.48
T_4_	37 (13.2)	14 (12.0)	23 (14.1)	0.72
Lymph node metastasis+	88 (31.4)	38 (32.5)	50 (30.7)	0.80
Distant tumor metastasis+	34 (12.1)	6 (5.1)	28 (17.2)	0.003
Medical insurance	209 (74.6)	83 (70.9)	126 (77.3)	0.266

Continuous data are reported as means ± standard deviations or medians (25th, 75th percentiles) and were compared using an independent *t* test or the Mann–Whitney *U* test, as appropriate. Categorical data are presented as numbers (proportions) and were compared using the Fisher exact test

PPLOS, prolonged postoperative length of stay; PLOS, postoperative length of stay; BMI, body mass index; ASA, American Society of Anesthesiologists; SpO_2,_ pulse oxygen saturation

**Table 2:** The associations between intraoperative variables and PPLOS

Characteristic	Total	PLOS ≥ 11 days		*P*-value
		Yes, *n* = 117	No, *n* = 163	
Duration of surgery (min)	213 ± 81	223 ± 81	206 ± 80	0.071
Duration of surgery ≥ 3 h	165 (58.9)	77 (65.8)	88 (54.0)	0.05
Chief surgeon (excellent/good)	142/138	51/66	91/72	0.052
Surgical approach (A/B/C)	63/33/184	18/23/76	45/10/108	0.001
Parks procedure or NOSES	24 (8.6)	8 (6.8)	16 (9.8)	
Right hemicolectomy	39 (13.9)	10 (8.5)	29 (17.8)	
Miles procedure	16 (5.7)	13 (11.1)	3 (1.8)	
Left hemicolectomy	17 (6.1)	10 (8.5)	7 (4.2)	
Sigmoidectomy	117 (41.8)	49 (41.9)	68 (41.7)	
Dixon procedure	67 (23.9)	27 (23.1)	40 (24.5)	
Additional operation	36 (12.9)	16 (13.7)	20 (12.3)	0.72
Active enterostomy	56 (20.0)	21 (17.9)	35 (21.5)	0.55
Intraoperative surgical events	34 (12.1)	24 (20.5)	10 (6.1)	< 0.001
Blood loss (mL)	50 (50, 100)	80 (50, 100)	50 (50, 100)	0.014
Blood loss ≥ 100 mL	115 (41.1)	58 (49.6)	57 (35.0)	0.019
Lung-protective ventilation	140 (50.0)	65 (55.5)	75 (46.0)	0.15
PRBC or FP infusion	20 (7.1)	12 (10.3)	8 (4.9)	0.102
Urine output (ml/kg/h)	2.0 (1.3, 3.3)	2.0 (1.3, 3.2)	2.1 (1.2, 3.5)	0.85
Lactic acid level (mmol/L)	0.69 ± 0.23	0.72 ± 0.20	0.67 ± 0.24	0.14
Hemoglobin level (g/L)	102 ± 18	102 ± 19	102 ± 16	0.98
Glucose level (mmol/L)	5.8 ± 1.0	5.8 ± 0.9	5.8 ± 1.0	0.69

Continuous data are reported as means ± standard deviations or medians (25th, 75th percentiles) and were compared using an independent *t* test or the Mann–Whitney *U* test, as appropriate. Categorical data are presented as numbers (proportions) and were compared using the Fisher exact test

PPLOS, prolonged postoperative length of stay; PLOS, postoperative length of stay; PRBC, packed red blood cell; FP, frozen plasma; NOSES, natural orifice specimen extraction surgery; Parks procedure, coloanal anastomosis; Dixon procedure, anterior rectal cancer resection; Miles procedure, abdominoperineal rectal cancer resection

Approach A (similar trends for PPLOS): Right hemicolectomy, Parks, or NOSES. Approach B (similar trends for PPLOS): Miles or left hemicolectomy. Approach C (similar surgical procedures): Dixon or sigmoidectomy. Intraoperative surgical events: positive anastomotic leakage test, reoperation or intestinal anastomosis twice, duration of non-pneumoperitoneum ≥ 2 h, or abdominal drainage ≥ 200 mL in the postoperative anesthesia care unit. Lung-protective ventilation: low tidal volume ventilation with an open-lung strategy

**Table 3:** The associations between postoperative characteristics and PPLOS

Characteristic	Total	PLOS ≥ 11 days		*P*-value
		Yes, *n* = 117	No, *n* = 163	
Hypotension^a^	59 (21.1)	22 (18.8)	37 (22.7)	0.46
Albumin infusion	208 (74.3)	98 (83.8)	110 (67.5)	0.002
Early ambulation^b^	72 (25.7)	16 (13.7)	56 (34.4)	< 0.001
Physiotherapy^c^ on PODs 0–5	5 (1.8)	2 (1.7)	3 (1.8)	1.00
PRBC and FP infusion	10 (3.6)	6 (5.1)	4 (2.4)	0.33
Drainage on PODs 0–3 (mL)	420 (259, 679)	430 (314, 780)	420 (210, 655)	0.037
Drainage on PODs 0–3 ≥ 300 mL	194 (69.3)	91 (77.8)	103 (63.2)	0.012
Perioperative surgical event^#^	203 (72.5)	97 (82.9)	106 (65.0)	0.001
Hemoglobin level on POD 1 (g/L)	106 ± 18	105 ± 18	106 ± 18	0.60^*^
Albumin level on POD 1 (g/L)	29.5 ± 3.9	29.1 ± 3.7	29.8 ± 4.0	0.16^*^
Albumin level on POD 3 (g/L)	32.0 ± 3.9	31.7 ± 4.1	32.3 ± 3.7	0.30^*^
WBC count on POD 1 (× 10^9^/L)	9.8 ± 3.0	10.0 ± 3.1	9.6 ± 2.9	0.29^*^
WBC count on POD 3 (× 10^9^/L)	8.8 ± 3.2	9.1 ± 3.2	8.7 ± 3.2	0.30^*^
hs-CRP level on POD 1 (mg/L)	68 (44, 92)	71 (40, 93)	68 (47, 91)	0.77^*^
hs-CRP level on POD 3 (mg/L)	108 (74, 152)	123 (83, 169)	103 (71, 137)	0.024^*^
Other events^d^	12 (4.3)	7 (6.0)	5 (3.1)	0.25

Continuous data are reported as means ± standard deviations or medians (25th, 75th percentiles) and were compared using an independent *t* test or the Mann–Whitney *U* test, as appropriate. Categorical data are presented as numbers (proportions) and were compared using the Fisher exact test

^*^Analyzed based on raw data (0–40% loss rate) without imputation. PPLOS, prolonged postoperative length of stay; PLOS, postoperative length of stay; POD, postoperative day; WBC, white blood cell; PRBC, packed red blood cell; FP, frozen plasma; hs-CRP, high-sensitivity C-reactive protein; MAP, mean arterial pressure

^#^Intraoperative surgical events (positive anastomotic leakage test, reoperation or intestinal anastomosis twice, duration of non-pneumoperitoneum ≥ 2 h, or abdominal drainage ≥ 200 mL in the postoperative anesthesia care unit) or abdominal drainage ≥ 300 mL in the first three days postoperatively

^a^Lowest postoperative MAP divided by the mean of three preoperative MAP values ≤ 70%

^b^Up and ambulatory within 2 days after surgery

^c^Except for infrared therapy which is a routine treatment after abdominal surgery in our center

^d^Gallstone, kidney stone, intraperitoneal hyperthermic perfusion chemotherapy, or radiofrequency ablation of hepatic metastasis

**Table 4:** Univariate and multivariate analysis to identify influencing factors for PPLOS

Factors^a^	Univariate OR (95% CI)	*P*-value	Multivariate OR (95% CI)	*P*-value
Preoperative SpO_2_		0.014		0.006
≥ 96%	1 (Reference)		1 (Reference)	
< 96%	2.47 (1.20–5.05)		3.09 (1.38–6.92)	
Distant tumor metastasis		0.004		0.031
No	1 (Reference)		1 (Reference)	
Yes	0.26 (0.10–0.65)		0.34 (0.13–0.91)	
Surgical approach		0.001		0.012
Approach C	1 (Reference)		1 (Reference)	
Approach A	0.57 (0.31–1.06)	0.074	1.69 (0.86–3.35)	0.131
Approach B	3.27 (1.47–7.26)	0.004	4.51 (1.67–12.18)	0.003
Perioperative surgical event^#^		0.001		0.009
No	1 (Reference)		1 (Reference)	
Yes	2.61 (1.46–4.65)		2.44 (1.25–4.76)	
Postoperative albumin infusion^*^		0.003		0.018
No	1 (Reference)		1 (Reference)	
Yes	2.49 (1.38–4.49)		2.19 (1.14–4.19)	
Postoperative early ambulation^*^		< 0.001		0.002
No	1 (Reference)		1 (Reference)	
Yes	0.30 (0.16–0.56)		0.35 (0.18–0.68)	

^*^Modifiable factor. PPLOS, prolonged postoperative length of stay; SpO_2,_ pulse oxygen saturation; OR, odds ratio; CI, confidence interval. Approach A: Parks procedure, right hemicolectomy, or natural orifice specimen extraction surgery. Approach B: Miles procedure or left hemicolectomy. Approach C: Dixon procedure or sigmoidectomy

^#^Intraoperative events (positive anastomotic leakage test, reoperation or intestinal anastomosis twice, duration of non-pneumoperitoneum ≥ 2 h, or abdominal drainage ≥ 200 mL in the postoperative anesthesia care unit) or abdominal drainage ≥ 300 mL in the first three postoperative days

^a^Two variables considered clinically relevant (ASA physical status and lung-protective ventilation) and nine variables with a *P*-value < 0.1 (preoperative SpO_2_, distant tumor metastasis, surgical approach, chief surgeon, duration of surgery ≥ 3 h, blood loss ≥ 100 mL, perioperative surgical events, postoperative albumin infusion, and postoperative early ambulation) were introduced into the multivariate logistic regression model. A forward stepwise elimination with thresholds of input *P* < 0.05 and output *P* < 0.1 was used to select variables in the final model (goodness of fit: Hosmer–Lemeshow test, χ^2^ = 3.463, *P* = 0.749)

Finally, six influencing factors were identified, as shown in Table 5: preoperative SpO_2_ < 96% (OR, 3.09 [95% CI 1.38–6.92]; *P* = 0.006), distant tumor metastasis (OR, 0.34 [95% CI 0.13–0.91]; *P* = 0.031), the Miles procedure or left hemicolectomy (OR, 4.51 [95% CI 1.67–12.18]; *P* = 0.003), perioperative surgical events (OR, 2.44 [95% CI 1.25–4.76]; *P* = 0.009), postoperative albumin infusion (OR, 2.19 [95% CI 1.14–4.19]; *P* = 0.018), and postoperative early ambulation (OR, 0.35 [95% CI 0.18–0.68]; *P* = 0.002). The goodness of fit of the final multiple regression model was good (Hosmer–Lemeshow test, χ^2^ = 3.463, *P* = 0.749).

**Table 5:** Stratified analysis of the associations between postoperative albumin infusion and PPLOS

	Postoperative albumin infusion		OR (95% CI)	*P*-value
	Yes	No		
	(PPLOS^+^/total [n/N])			
Preoperative albumin level				
< 40 g/L	54/117	9/33	2.29 (0.98–5.34)	0.056
≥ 40 g/L	44/91	10/39	2.72 (1.19–6.21)	0.018
Duration of surgery				
≥ 3 h	68/134	9/31	2.52 (1.08–5.87)	0.032
< 3 h	30/74	10/41	2.11 (0.90–4.95)	0.085

Categorical data are presented as numbers and were compared using univariate logistic regression analysis

PPLOS, prolonged postoperative length of stay; OR, odds ratio; CI, confidence interval

Postoperative albumin infusion was not related to preoperative nutritional status (preoperative albumin level, preoperative hemoglobin level, age, BMI, ASA physical status, radiotherapy, chemotherapy, and diabetes mellitus; all, *P* ≥ 0.05), but it was related to surgical factors (duration of surgery ≥ 3 h, blood loss ≥ 100 mL, and perioperative surgical events) (see Additional file 1: Table S1). Further stratified analysis showed that postoperative albumin infusion might be associated with PPLOS, even in patients with a preoperative albumin level < 40 g/L (OR, 2.29 [95% CI 0.98–5.34]; *P* = 0.056) or duration of surgery ≥ 3 h (OR, 2.52 [95% CI 1.08–5.87]; *P* = 0.032) (Table 5).

## Discussion

This study showed that a preoperative SpO_2_ < 96%, the Miles procedure or left hemicolectomy, perioperative surgical events, postoperative albumin infusion, distant tumor metastasis, and postoperative early ambulation were associated with PPLOS after laparoscopic colorectal cancer resection. We further found that postoperative albumin infusion was an independent and modifiable risk factor for PPLOS. This study is based on the data of a randomized trial; therefore, it was possible to comprehensively evaluate the association between perioperative factors (baseline, anesthesia, surgery, and postoperative management) and PPLOS.

We found that 38.5% of the patients in the case group had postoperative surgical complications (see Additional file 1: Table S2), that longer duration of surgery, perioperative surgical events, more intraoperative blood loss, and a suboptimal chief surgeon were all related to PPLOS than in their counterparts (Tables 3, 4), suggesting that surgical factors were the main influencing factors for PPLOS after laparoscopic colorectal cancer resection. However, multivariate analysis found that other factors were also related to PPLOS. It indicated that PPLOS resulted from a combination of surgical and multiple factors [14, 16]. Herein, variables related to anesthesia, such as intraoperative lung-protective ventilation, urine volume, and lactic acid level, were not associated with PPLOS. This may be related to the fact that the sample size of this study was still small; it also suggested that anesthetic factors might have less effect on PLOS than surgical factors and the patients’ baseline characteristics.

Inconsistent with previous studies [14, 15], we did not find an association between ASA physical status and PPLOS. We reasoned that the sample size was small, and only patients with an ASA physical status of II–III were enrolled in the study. In the present study, the incidence of postoperative pulmonary complications was higher and the median PLOS was longer than that reported previously [7, 17]. This discrepancy may be related to the included older patients (mean age: 70.3 years) with an intermediate-to-high risk for postoperative pulmonary complications.

A low preoperative pulse oximetry reading was a risk factor, whereas age was not associated with PPLOS in this study. A possible explanation is that there is an interaction between the heart and lung, and damage to both organs could lead to a decrease in SpO_2_ [18]. These results suggest that cardiorespiratory status, rather than age, has an important effect on PLOS [15, 16]. We found that PPLOS was more likely to occur in patients undergoing the Miles procedure or left hemicolectomy than in those undergoing other surgical procedures. This might be related to more complex surgical procedures and greater trauma in these two procedures compared with other surgical procedures [16, 17].

We found that distant tumor metastasis was a protective factor against PPLOS. We speculate that this may be related to the conservative surgical strategies used on these patients so that they tend to have a shorter operation time, less bleeding and fewer intraoperative surgical events though there was no statistical difference (see Additional file 1: Table S3). However, we should be cautious about this result because it resulted from a special population (older age with a risk for pulmonary complications) and was inconsistent with the results of a previous study with a large sample size [19]. ERAS pathways have been widely implemented in colorectal surgery to reduce physiological stress induced by surgery and postoperative complications [1, 20] and are associated with a significantly reduced PLOS in many different areas of surgery [6, 21]. Herein, postoperative early ambulation, as an important strategy for ERAS, was found to be a protective factor for PPLOS after laparoscopic colorectal cancer resection, which further demonstrates the importance of ERAS in promoting early recovery.

Postoperative hypoalbuminemia is often considered to be related to poor prognosis [22, 23]. Postoperative albumin infusion is usually used to reduce tissue edema, improve wound healing, and reduce complications [24, 25]. These considerations were also reflected in this study (see Additional file 1: Table S1). However, we found that postoperative albumin infusion was a risk factor for PPLOS in patients with preoperative hypoalbuminemia and in those with a longer duration of surgery (Table 5). Numerous studies [22, 26] have supported this conclusion and found that postoperative albumin infusion did not improve clinical outcomes and that albumin infusion was positively correlated with surgical complications, peritoneal hemorrhage, and pancreatic fistula. We reasoned that postoperative albumin infusion could neither increase the plasma albumin level to normal (see Additional file 1: Table S1) nor change the comprehensive nutritional status of the body. On the contrary, exogenous albumin infusion disrupts the nutritional balance of the body and increases the risk of adverse reactions, e.g., allergies. Therefore, postoperative albumin infusion may be a risk factor. However, this result needs to be further verified in larger samples, especially in prospective clinical trials. Since postoperative albumin infusion is a common and modifiable postoperative management behavior, and if it can be determined as a risk factor, changing this clinical behavior would improve the clinical outcomes of patients and decrease medical costs. Therefore, this study and any related clinical research that will be conducted in the future have important clinical significance.

This study had several limitations. First, the study population was patients undergoing laparoscopic colorectal cancer resection; therefore, the external reliability of its conclusions needs to be further verified. However, the incidence of colorectal cancer is high; therefore, studies on the factors influencing PPLOS in this population also have very important clinical significance. Second, the PROVOLON trial was not specifically designed to study PLOS, which may have led to differences in the postoperative discharge criteria. Nevertheless, our unit is well known for colorectal cancer surgery, and bed resources are limited; thus, we believe that subjective factors had little effect on PLOS in this study. Third, the diagnosis of postoperative complications might have been missed in 20 patients due to missing follow-up data in the PROVOLON trial. However, postoperative complications were not the primary outcome of this study. In addition, all postoperative complications in this study were re-diagnosed using postoperative follow-up data, medical records, imaging findings, and laboratory test results. Lastly, the sample size of this case–control study was small. However, this study was a secondary analysis of a prospective randomized controlled trial with detailed data; hence, its reliability is still high.

## Conclusions

In conclusion, a low preoperative pulse oximetry reading, complex surgical procedures, postoperative albumin infusion, and perioperative surgical events may be risk factors for PPLOS after laparoscopic colorectal cancer resection, whereas postoperative early ambulation and distant tumor metastasis might be protective factors. Further studies on the association between postoperative albumin infusion and PLOS or clinical outcomes are warranted and meaningful.

## Supplementary Information

**Additional file 1**: **Table S1. **The associations between clinical characteristics and postoperative albumin infusion. **Table S2.** Postoperative complications. **Table S3. **Operation and tumor metastasis.

## Data Availability

The data for this study are available from the corresponding author on reasonable request.

## Abbreviations

LOS: Length of hospital stay
PLOS: Postoperative length of hospital stay
ERAS: Enhanced recovery after surgery
PPLOS: Prolonged postoperative length of hospital stay
PROVOLON: PROtective Ventilation using Open Lung approach Or Not
BMI: Body mass index
ASA: American Society of Anesthesiologists
MAP: Mean arterial pressure
CRP: C-reactive protein
SpO_2_: Pulse oxygen saturation
OR: Odds ratio
CI: Confidence interval
