# Supplementary material for: Factors associated with prolonged postoperative length of hospital stay after laparoscopic colorectal cancer resection: a secondary analysis of a randomized controlled trial
Source: BMC Surg. 2022 Dec 24;22:438. doi: 10.1186/s12893-022-01886-4 (PMC9789636; doi:10.1186/s12893-022-01886-4)
Supplement: Supplementary file 1 — Additional file 1: Table S1. The associations between clinical characteristics and postoperative albumin infusion. Table S2. Postoperative complications. Table S3. Operation and tumor metastasis. [file 12893_2022_1886_MOESM1_ESM.docx]

**Table S1** The associations between clinical characteristics and postoperative albumin infusion

| Characteristics | Postoperative albumin infusion | | *P*-value | |  |
| --- | --- | --- | --- | --- | --- |
|  | Yes, *n* = 208 | No, *n* = 72 | |  | |
| PLOS (days) | 10 (8, 13) | 9 (7, 11) | | 0.002 | |
| PPLOS (%) | 98 (47.1) | 19 (26.4) | | 0.002 | |
| Preoperative albumin level (g/L) | 39.1 ± 4.0 | 39.9 ± 3.0 | | 0.057 | |
| <35 g/L | 32 (15.4) | 6 (8.3) | | 0.163 | |
| <40 g/L | 117 (56.3) | 33 (45.8) | | 0.134 | |
| Age (years) | 70.3 ± 5.7 | 70.3 ± 6.0 | | 0.98 | |
| Body mass index (kg/m^2^) | 22.62 ± 2.71 | 22.72 ± 2.96 | | 0.80 | |
| Preoperative hemoglobin level (g/L) | 120 ± 20 | 125 ± 21 | | 0.09 | |
| ASA physical status (II/III) | 164/44 | 62/10 | | 0.23 | |
| Body weight loss ≥10% | 44 (21.2) | 17 (23.6) | | 0.74 | |
| Radiotherapy | 19 (9.1) | 3 (4.2) | | 0.21 | |
| Chemotherapy | 33 (15.9) | 8 (11.1) | | 0.44 | |
| Diabetes mellitus | 27 (13.0) | 7 (9.7) | | 0.54 | |
| Duration of surgery (min) | 221 ± 81 | 191 ± 77 | | 0.006 | |
| Blood loss ≥100 mL | 97 (46.6) | 18 (25.0) | | 0.001 | |
| Perioperative surgical events^#^ | 161 (77.4) | 42 (58.3) | | 0.003 | |
| Drainage ≥300 ml on PODs 0–3 | 154 (74.0) | 40 (55.6) | | 0.005 | |
| Albumin level on POD 1 (g/L) | 29.0 ± 3.5 (*n*=193) | 30.9 ± 4.7 (*n*=57) | | 0.001 | |
| Albumin level on POD 3 (g/L) | 32.6 ± 3.9 (*n*=162) | 30.1 ± 3.0 (*n*=49) | | <0.001 | |

Continuous data are reported as means ± standard deviations or medians (25th, 75th percentiles) and were compared using an independent *t* test or the Mann–Whitney *U* test, as appropriate. Categorical data are presented as numbers (proportions) and were compared using the Fisher exact test.

PPLOS, prolonged postoperative length of stay; PLOS, postoperative length of stay; POD, postoperative day; ASA, American Society of Anesthesiologists

^#^ Intraoperative surgical events (positive anastomotic leakage test, reoperation or intestinal anastomosis twice, duration of non-pneumoperitoneum ≥2 h, or abdominal drainage ≥200 mL in the postoperative anesthesia care unit) or abdominal drainage ≥300 mL in the first three days postoperatively.

**Table S2** Postoperative complications

| Postoperative complications | Total (%) | PLOS ≥11 days | |
| --- | --- | --- | --- |
|  |  | Yes, *n*=117 | No, *n*=163 |
| One or more complications | 94 (33.6) | 63 (53.8) | 31 (19.0) |
| Clavien-Dindo classification^[1]^ (I) | 40 (14.3) | 17 (14.5) | 23 (14.1) |
| Clavien-Dindo classification (II) | 39 (13.9) | 32 (27.4) | 7 (4.3) |
| Clavien-Dindo classification (III) | 13 (4.6) | 12 (10.3) | 1 (0.6) |
| Clavien-Dindo classification (IV) | 2 (0.7) | 2 (1.7) | 0 |
| Surgical complications | 49 (16.1) | 45 (38.5) | 4 (2.5) |
| Bleeding | 5 (1.8) | 3 (2.6) | 2 (1.2) |
| Anastomotic leakage^[2]^ (C) | 5 (1.8) | 4 (3.4) | 1 (0.6) |
| Anastomotic leakage (B) | 6 (2.1) | 6 (5.1) | 0 |
| Anastomotic leakage (A) | 8 (2.9) | 7 (6.0) | 1 (0.6) |
| Abdominal abscess | 13 (4.6) | 12 (10.3) | 1 (0.6) |
| Intestinal obstruction | 7 (2.5) | 7 (6.0) | 0 |
| Poor wound healing | 7 (2.5) | 7 (6.0) | 0 |
| Abdominal distension for 1 week | 4 (1.4) | 4 (3.4) | 0 |
| Unexplained leukocytosis | 2 (0.7) | 2 (1.7) | 0 |
| Urinary retention | 4 (1.4) | 4 (3.4) | 0 |
| Ureteral fistula | 2 (0.7) | 2 (1.7) | 0 |
| Incarcerated necrosis of small intestine | 1 (0.4) | 1 (0.9) | 0 |
| Pleural effusion needs drainage | 1 (0.4) | 1 (0.9) | 0 |
| Pneumonia or suspected pneumonia^[3]^ | 20 (7.1) | 16 (13.7) | 4 (2.5) |
| Respiratory failure^[3, 4]^ | 65 (23.2) | 38 (32.5) | 27 (16.6) |
| Cardiac insufficiency | 1 (0.4) | 1 (0.9) | 0 |

Data are presented as numbers (proportions); PLOS, postoperative length of stay

1. Dindo D, Demartines N, Clavien PA. Classification of surgical complications: a new proposal with evaluation in a cohort of 6336 patients and results of a survey. Ann Surg*.* 2004;240:205-13.

2. Rahbari NN, Weitz J, Hohenberger W, Heald RJ, Moran B, Ulrich A, et al. Definition and grading of anastomotic leakage following anterior resection of the rectum: a proposal by the International Study Group of Rectal Cancer. Surgery*.* 2010;147:339-51.

3. Li H, Zheng ZN, Zhang NR, Guo J, Wang K, Wang W, et al. Intra-operative open-lung ventilatory strategy reduces postoperative complications after laparoscopic colorectal cancer resection: a randomised controlled trial. Eur J Anaesthesiol*.* 2021;38:1042-51.

4. Jammer I, Wickboldt N, Sander M, Smith A, Schultz MJ, Pelosi P, et al. Standards for definitions and use of outcome measures for clinical effectiveness research in perioperative medicine: European Perioperative Clinical Outcome (EPCO) definitions: a statement from the ESA-ESICM joint taskforce on perioperative outcome measures. Eur J Anaesthesiol*.* 2015;32:88-105.

**Table S3** Operation and tumor metastasis

| Characteristics | Distant tumor metastasis | | *P*-value |
| --- | --- | --- | --- |
|  | Yes, *n*=34 | No, *n*=246 |  |
| Duration of surgery (min) | 201 ± 88 | 215 ± 80 | 0.342 |
| Duration of surgery ≥3 h | 16 (47.1) | 149 (60.6) | 0.142 |
| Intraoperative surgical events | 1 (2.9) | 33 (13.4) | 0.095 |
| Blood loss (mL) | 50 (50, 100) | 50 (50, 100) | 0.317 |
| Blood loss ≥100 mL | 11 (32.4) | 104 (42.3) | 0.353 |
| Dissected lymph nodes^a^ (No.) | 19 ± 8 | 17 ± 8 | 0.167 |

Continuous data are reported as means ± standard deviations or medians (25th, 75th percentiles) and were compared using an independent *t* test or the Mann–Whitney *U* test, as appropriate. Categorical data are presented as numbers (proportions) and were compared using the Fisher exact test.

Intraoperative surgical events: positive anastomotic leakage test, reoperation or intestinal anastomosis twice, duration of non-pneumoperitoneum ≥2 h, or abdominal drainage ≥ 200 mL in the postoperative anesthesia care unit

^a^ The data are from the Cancer Database of The Sixth Affiliated Hospital, Sun Yat-sen University, Guangzhou, China
